# Hospital Annual Meetings

**Published:** 1919-04-12

**Authors:** 


### HOSPITAL ANNUAL MEETINGS.

### Middlesex Hospital.

The annual meeting was presided over by Mr. F. Cavendish Bentinck, deputy-chairman of the Board, who said the income during the past year was £50,180—less than the expenditure by over £4,000. The strain of the war had been especially felt by the nurses, for whom Princess Alice, Countess of Athlone, had raised a fund in order to provide better and improved accommodation. The general renovation of the electrical apparatus and hot-water supply would take place this year and necessitate the closing of the hospital during the months of July, August, and September. Cancer cases during that period would be removed to Clacton-on-Sea. The arrangement with the Ministry of Pensions for the free treatment of discharged soldiers and sailors would, he was able to announce, be continued for another year. It was expected that the present Duke of Northumberland would consent to become President, in succession to his father, who filled the post for nearly twenty years.

### Leicester Royal Infirmary.

"To deal with the business arising from an agenda which includes eighteen speakers in an hour and a quarter and to finish exactly to time is testimony to the businesslike methods of the Leicester Royal Infirmary," said the Bishop of Peterborough in presiding at the 146th annual meeting on Wednesday. And it may be said that in no year has a more successful meeting been held or under a more businesslike president.

Mr. T. Fielding Johnson, junior, the Chairman of the Board, in moving the adoption of the report, stated that, comparing the figures of 1914 with 1918, annual subscriptions had been doubled at £9,000, due to the Treasurer's great effort; investments had increased from £59,000 to £93,000; after writing off depreciation, £5,000 had been secured towards the new pathological department; and £14,000 obtained towards the cost of the Sir Edward Wood Memorial Wing. In addition, he was proud to say that the Freemasons of Leicestershire had selected the proposed orthopædic out-patients department at the institution as their war memorial. The pathological department and new mortuary had already been started, and they hoped in the course of a month to be in a position to invite tenders for the orthopædic department. The Board also had under consideration the establishment of a paying ward or wards, which would be a great asset to the county, and an isolation hospital, the need for which had often been felt by the hon. medical staff. He paid a glowing tribute to the staff and nurses of the institution during a most trying and difficult period. The Bishop of Peterborough, in supporting the resolution, referred to the work of the medical institutions of the country in the direction of prevention of disease. His Lordship hoped that the University College proposed for the county of Leicester would be cordially supported, for he felt that from every point of view a spread of education would be highly necessary, and would in his opinion do much to prevent the causes of industrial trouble in itself partly due to lack of knowledge. Major H. G. Blakesley, on his retirement from the senior surgeoncy, was elected a consulting surgeon.

### The Italian Hospital.

Cav. Preziosi, Councillor of the Italian Embassy, occupied the chair at the annual meeting. Sir Stuart Coats, M.P., chairman of the hospital, said that since the outbreak of hostilities to the end of last year the institution had treated 1,704 military patients. It had continued the examination of recruits for the Italian Army, and its services in that direction had been recognised by the Italian Government in the shape of various decorations. Lord Edmund Talbot, M.P., said that signs were appearing on the horizon where hospitals were concerned which he viewed with some apprehension. The advancement of science necessitated, it was said, more constant individual attention on the part of doctors. Means of support other than voluntary ones would, if that were so, have to be sought by hospitals. He would regret to see the management of voluntary institutions handed over to the State.

### Brompton Hospital for Consumption.

The annual Court was presided over by the Duke of Wellington. The report stated that a large number of cases had passed through the wards, most of whom it had been found possible to transfer to the Sanatorium at Frimley for a prolonged term of treatment by the special system of graduated exercises and work which has proved so successful at that institution. The average stay of these patients was approximately six months. In response to a special appeal the Committee have been able to acquire the further land required for the full development of the sanatorium, and they are now in a position to make an addition of 100 to 150 beds whenever required. This project is viewed favourably by the authorities in connection with the provision of treatment for men discharged from the Services, and the Committee are making a special appeal for funds in order to enable them to provide such extra beds as may be needed forthwith.

Both sisters' and nurses' salaries have been increased and the hours on duty reduced by increasing the nursing staff.

### Hospital for Consumption, Ventnor.

Lord Blyth, presiding over the annual Court of this Isle of Wight institution, held at 33 Portland Place, said last year 803 patients were cared for, as compared with 689 in 1917. The first want of the hospital was a nurses' home, which would permit of part of the hospital building now being used for the housing of the nursing staff to be released for the extra accommodation of patients. Although the hospital had managed to keep out of debt during the war, there was little doubt that a further income of £5,000 a year would be required to ensure that state of affairs in the future. The total annual income required was between £20,000 and £25,000. Dr. Robert Robertson, one of the hospital's physicians said since 1877 mortality from consumption at Ventnor had been reduced by 84 per cent.

### Hospital for Women, Soho.

Earl Winterton, M.P., occupied the chair at the annual Court. The hospital last year cared for 1,020 in-patients as compared with 996 in 1917. Out-patients last year numbered 3,760, with 12,106 attendances, as against 2,765 and 8,601 attendances a year previously. The Samaritan Fund enabled 66 persons to be sent to the convalescent homes. Expenditure reached the record figure of £9,029, as compared with £7,715 in 1917. Total income was £9,354, thus showing a balance on the credit side of £325. The average cost of each in-patient per week in 1918 was £2 10s., and of each out-patient 4s. 9d.

### London Lock Hospital.

It was stated at the annual meeting, held under the presidency of Lord Kinnaird, that since June 68 maternity cases had been treated and 53 babies born. At least £1,200 a year was required for the upkeep of that work. The total expenditure for 1918 of both the male and female hospital and the rescue home was £24,804, while income only amounted to £21,658. Salaries of ward sisters had been increased to £50 a year, rising to £60. A centre for the training of midwives had been established.

### London Temperance Hospital.

At the annual meeting, under the presidency of Sir Vezey Strong, emphasis was laid upon the need for increased financial support. The declining income of the institution is causing the Board grave concern, the deficiency last year having amounted to about £3,200. General annual expenditure amounted to about £14,000, while reliable income only totalled £6,000. Last year 1,154 in-patients were cared for, making 41,467 since the hospital was established forty-six years ago. Out-patients and casualties numbered 15,803, with 68,570 attendances—a substantial increase as compared with 1917. Soldiers received in the wards last year numbered 138.

### National Orthopædic Hospital.

The Earl of Denbigh presided over the annual meeting of the Royal National Orthopædic Hospital, which during the greater part of last year has set aside 150 of its 200 beds for the use of men discharged from the Services. As from the beginning of March, however, fifty more beds have been released for civilian patients. The civil in-patients last year only numbered 299; out-patients totalled 48,728. Total income during 1918 was £22,809, while expenditure in the same period amounted to £22,198. The hospital is affording training in the making of surgical boots and appliances for discharged men.

